# *TERT* Promoter Mutations Are Frequent in Cutaneous Basal Cell Carcinoma and Squamous Cell Carcinoma

**DOI:** 10.1371/journal.pone.0080354

**Published:** 2013-11-18

**Authors:** Klaus G. Griewank, Rajmohan Murali, Bastian Schilling, Tobias Schimming, Inga Möller, Iris Moll, Marion Schwamborn, Antje Sucker, Lisa Zimmer, Dirk Schadendorf, Uwe Hillen

**Affiliations:** 1 Department of Dermatology, University Hospital, University Duisburg-Essen, Essen, Germany; 2 Department of Pathology, Memorial Sloan-Kettering Cancer Center, New York, United States of America; 3 Human Oncology and Pathogenesis Program, Memorial Sloan-Kettering Cancer Center, New York, United States of America; Ohio State University Medical Center, United States of America

## Abstract

Activating mutations in the *TERT* promoter were recently identified in up to 71% of cutaneous melanoma. Subsequent studies found *TERT* promoter mutations in a wide array of other major human cancers. *TERT* promoter mutations lead to increased expression of telomerase, which maintains telomere length and genomic stability, thereby allowing cancer cells to continuously divide, avoiding senescence or apoptosis. *TERT* promoter mutations in cutaneous melanoma often show UV-signatures. Non-melanoma skin cancer, including basal cell carcinoma and squamous cell carcinoma, are very frequent malignancies in individuals of European descent. We investigated the presence of *TERT* promoter mutations in 32 basal cell carcinomas and 34 cutaneous squamous cell carcinomas using conventional Sanger sequencing. *TERT* promoter mutations were identified in 18 (56%) basal cell carcinomas and in 17 (50%) cutaneous squamous cell carcinomas. The recurrent mutations identified in our cohort were identical to those previously described in cutaneous melanoma, and showed a UV-signature (C>T or CC>TT) in line with a causative role for UV exposure in these common cutaneous malignancies. Our study shows that *TERT* promoter mutations with UV-signatures are frequent in non-melanoma skin cancer, being present in around 50% of basal and squamous cell carcinomas and suggests that increased expression of telomerase plays an important role in the pathogenesis of these tumors.

## Introduction

Non-melanoma skin cancers including basal cell carcinoma (BCC) and squamous cell carcinoma (SCC) are the most frequent tumors in individuals of European descent [1], [2]. Their incidences have increased considerably which is attributed to the aging population and high levels of sun exposure [3], [4].

BCC is the most frequent type of skin cancer. Established risk factors for BCC include fair skin, blond or red hair, light eye color [5], UV-exposure, radiation, and immunosuppression [3], [5]. BCCs harbor mutations leading to activation of the hedgehog pathway [3]. Inactivating mutations in the tumor suppressor *PTCH1* were first identified in patients with basal cell nevus syndrome, [6], [7] and subsequently reported in up to 90% of sporadic BCC [3], [8], [9]. Activating mutations in other hedgehog pathway genes, such as *SMO*, *SHH*, or *GLI*, are found less frequently (∼10% of tumors) [10]. BCC were also shown to harbor frequent UV-signature mutations in *TP53* [11], [12]. Recently novel therapies inhibiting the hedgehog signaling pathway have shown high efficacy in treating patients with inoperable or metastatic BCC [13].

SCC is less frequent than BCC (ratio circa 1∶4) [4]. UV-exposure is considered to be the most important etiologic factor in SCC [2]. SCC has the potential to metastasize, and may result in death, particularly in organ transplant recipients [14]. The prognosis for metastatic SCC is poor, with ten-year survival rates of less than 10% [4]. Genetic alterations in SCCs include activating mutations or gains of *RAS* genes [15], as well as mutations in *TP53*, frequently with a C>T or CC>TT UV-signature [16]. Other genetic events are overexpression or mutations of *EGFR*, losses of *CDKN2A* [17], and inactivating mutations of *NOTCH* [18].

Novel mutations in the promoter region of *TERT*, coding for the catalytic subunit of the telomerase holoenzyme, were identified in up to 71% of cutaneous melanomas in two recent studies [19], [20]. These mutations lead to increased *TERT* expression, most likely by creating ETS transcription factor binding sites [19], [20]. *TERT* promoter mutations were also found in a number of other common cancers, including hepatocellular cancer, bladder cancer, thyroid cancer and gliomas [21]–[24]. In cutaneous tumors, *TERT* promoter mutations with high numbers of UV-signature mutations were also identified in atypical fibroxanthomas and pleomorphic dermal sarcomas, rare soft tissue tumors arising in heavily UV-damaged skin [25]. One current study reported a *TERT* promoter mutation in one of five SCCs [21], and another recent publication found *TERT* promoter mutations in 78% of BCC and 50% of SCC [26].

In our study we investigate the presence of *TERT* promoter mutations in BCCs and SCCs, and their associations with clinical and pathologic features.

## Materials and Methods

### Sample selection

Samples of primary BCCs and SCCs were obtained from 66 patients treated in the Department of Dermatology, University Hospital Essen, Germany. The study was approved by the Institutional Review Board of the University of Duisburg-Essen (*Ethikkomission der Universität Duisburg-Essen*) under the IRB protocol number 12-4961-BO. All patients provided written informed consent.

### DNA isolation

10 µm-thick sections were cut from formalin-fixed, paraffin-embedded tumor tissues. The sections were deparaffinized and tumor tissue was manually macrodissected. Genomic DNA was isolated using the QIAamp DNA Mini Kit (Qiagen, Hilden, Germany) according to the manufacturer's instructions.

### Direct (Sanger) sequencing

PCR amplification of the *TERT* promoter region was performed using primers: hTERT_F ACGAACGTGGCCAGCGGCAG and hTERT_R CTGGCGTCCCTGCACCCTGG (474 bp product), or primers hTERT_short_F CAGCGCTGCCTGAAACTC and hTERT_short_R GTCCTGCCCCTTCACCTT (163bp product) as previously described [20]. PCR products were used as templates for sequencing after purification with the QIAquick PCR Purification Kit (Qiagen). Sequencing chromatogram files were examined using Chromas (version 2.01, University of Sussex, Brighton, United Kingdom) or Sequencher (demo version 5.1, Gene Codes Corporation, Ann Arbor, MI, USA) software.

## Results

### Study cohort

The study cohort consisted of 32 BCC and 34 SCC samples, from 43 males and 23 females. The median age was 72.6 years for SCC and 73.0 years for BCC. Histopathologic analysis was performed on all samples to assess histologic subtype, tumor thickness, cystic component, ulceration status, and presence of pigmentation for BCC, as well as tumor thickness, Clark level, acantholysis, lymphovascular involvement (LVI), perineural involvement (PNI), and presence of ulceration in SCC. Clinicopathologic characteristics are listed in Table 1.

**Table 1:** *TERT* promoter mutations identified in BCC and SCC.

	BCC	SCC
Wild-type	14 (44%)	17 (50%)
Mutant	18 (56%)	17 (50%)
*Mutations*		
*c.-146C>T*	*10 (31%)*	*5 (15%)*
*c.-124C>T*	*4 (13%)*	*5 (15%)*
*c.-138_139CC>TT*	*2 (6%)*	*4 (12%)*
*c.-124_125CC>TT*	*1 (3%)*	*2 (6%)*
*c.-124C>T, c.-126C>T*	*0*	*1 (3%)*
*c.-126_127CC>TT, c.-146C>T*	*1 (3%)*	*0*

### *TERT* promoter mutation analysis

Recurrent TERT promoter mutations were identified in both BCC and SCC. Mutations were located at Chr.5.1295228C>T, Chr.5.1295228_1295229CC>TT, Chr.5.1295242_1295243CC>TT, or Chr.5.1295250C>T. Alternatively mutations may be denoted based on their upstream location to the ATG initiation codon of *TERT*, as c.-124C>T, c.-124_125CC>TT, c.-138_139CC>TT, and c.-146C>T, respectively. For the rest of the manuscript we will refer to the mutations using this annotation.

**Figure 1:**
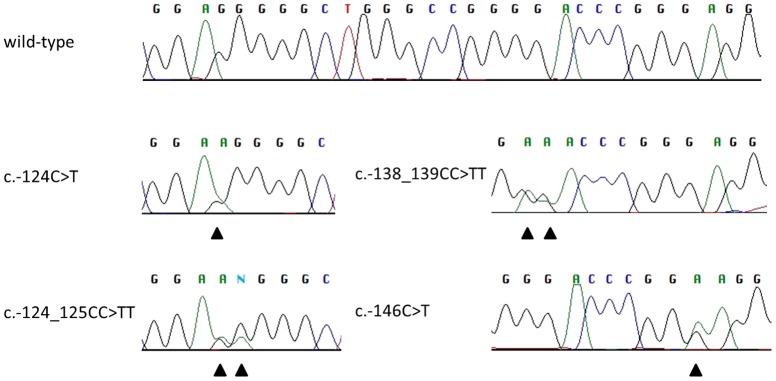
Recurrent mutations identified in the *TERT* promoter of BCC and SCC. Representative sequencing chromatograms showing the wild type sequence (on top) and representative examples of the mutations identified in both basal and squamous cell carcinoma samples – c.-124C>T, c.-124_125CC>TT, c.-138_139CC>TT or c.-146C>T (alternatively annotated according to the chromosome location as Chr.5. 1295228C>T, Chr.5. 1295228_1295229CC>TT, Chr.5. 1295242_1295243CC>TT or Chr.5.1295250C>T, respectively). All presented mutations were found in both tumor cohorts however the presented chromatograms are from a BCC, SCC, BCC, and BCC, respectively.

In BCC, *TERT* promoter mutations were identified in 18 (56%) cases (Table 1). Recurrent mutations were located at positions c.-124C>T (n = 4, 13%), c.-124_125CC>TT (n = 1, 3%), c.-138_139CC>TT (n = 2, 6%), or c.-146C>T (n = 11, 34%), as shown in Figure 1. One c.-146C>T mutant tumor also harbored a c.-126_127CC>TT mutation. Seventeen (50%) SCCs harbored *TERT* promoter mutations (Table 1), which included c.-124C>T (n = 6, 18%), c.-124_125CC>TT (n = 2, 6%), c.-138_139CC>TT (n = 4, 12%), and c.-146C>T (n = 5, 15%). One c.-124C>T case had a concomitant c.-126C>T mutation. All identified mutations showed a UV-signature (C>T and CC>TT) [27].

### Associations of clinical and pathologic parameters with *TERT* promoter mutation status

Apart from a small, statistically significant (p = 0.046) difference in age between patients with *TERT* promoter-mutant BCCs (median 75.5 years) and those with *TERT* promoter-wild type BCCs (median 71.0 years), there were no statistically significant associations of *TERT* mutation status with clinicopathologic parameters (Table 2 and 3).

**Table 2:** Associations of clinical and pathologic parameters in BCC with *TERT* promoter mutation status.

Parameter	Level	All cases	*TERT^wt^*	*TERT^mut^*	P value**
		N = 32	N = 14	N = 18	
Age at diagnosis	Median	73	71.0	75.5	0.046
(years)	Range	30–90	30–87	52–90	
Sex	Female	10	4	6	1.00
	Male	22	10	12	
Tumor location	Head & neck	23	10	13	0.36
	Extremities	2	0	2	
	Trunk	7	4	3	
Tumor thickness	Median	1.2 mm	1.1 mm	1.35 mm	0.16
	Range	0.6–4.1 mm	0.6–2.8 mm	0.8–4.1 mm	
Histologic type	Nodular	15	7	8	0.20
	Micronodular	5	1	4	
	Superficial	8	5	3	
	Infiltrative	3	0	3	
	Infundibulocystic	1	1	0	
Cystic component	No	24	10	14	0.70
	Yes	8	4	4	
Ulceration	No	17	10	7	0.07
	Yes	15	4	11	
Pigment	No	31	14	17	1.00
	Yes	1	0	1	

*TERT^wt^*  =  *TERT* promoter wild-type; *TERT^mut^*  =  *TERT* promoter mutant.

** Based on chi-squared or Fisher exact tests for categorical variables, and on Mann-Whitney test for continuous variables. Cases with missing data were excluded from statistical analyses.

Histologic parameters analyzed were based on the World Health Organization's classification and histologic criteria[31]. Tumor thickness was measured as for cutaneous melanomas[32].

**Table 3:** Associations of clinical and pathologic parameters in SCC with *TERT* mutation status.

Parameter	Level	All cases	*TERT^wt^*	*TERT^mut^*	P value**
		N = 34	N = 17	N = 17	
Age at diagnosis	Median	72.6	76.8	72.5	0.69
(years)	Range	46–95	46–90	52–95	
Sex	Female	13	7	6	0.72
	Male	21	10	11	
Tumor location	Head & neck	25	11	14	0.80
	Extremities	5	3	2	
	Trunk	2	1	1	
	Missing data	2	2	0	
Tumor thickness	Median	3.95 mm	4.7 mm	3.9 mm	0.95
	Range	2.8–10.0 mm	2.8–10.0 mm	3.0–10.0 mm	
Clark level	III	6	5	1	0.13
	IV	17	6	11	
	V	10	5	5	
Grade	1 (well differentiated)	8	4	4	0.72
	2 (moderately differentiated)	16	7	9	
	3 (poorly differentiated)	10	6	4	
Acantholysis	No	29	15	14	1.00
	Yes	5	2	3	
Ulceration	No	19	8	11	0.30
	Yes	15	9	6	
Desmoplasia	No	30	14	16	0.48
	Yes	3	2	1	
	Missing data	1	1	0	
Perineural invasion	No	28	15	13	0.66
	Yes	6	2	4	
Lymphovascular	No	33	16	17	1.00
invasion	Yes	1	1	0	
Mean survival			42.5	68.6	0.51*
(months)*			(33.7–51.4)	(57.5–79.7)	

*TERT^wt^*  =  *TERT* promoter wild-type; *TERT^mut^*  =  *TERT* promoter mutant.

* Estimates of mean survival from Kaplan Meier method (median survival not reached); p value estimated using log-rank test.

** Based on chi-squared or Fisher exact tests for categorical variables, and on Mann-Whitney test for continuous variables. Cases with missing data were excluded from statistical analyses.

Histologic parameters analyzed were based on the World Health Organization's classification and histologic criteria[33]. Clark level of invasion and tumor thickness were measured as for cutaneous melanomas[32].

## Discussion

BCC and SCC harbor distinct patterns of genetic alterations. BCC have genetic alterations activating the hedgehog signaling pathway. In contrast, SCC show alterations leading to activation of the MAPK and AKT signaling pathway, such as overexpression or mutations of genes such as *RAS*, *EGFR*, or *PIK3CA* [17], [18]. Losses of *CDKN2A* [17] and inactivation of *NOTCH* [18] are also frequent in SCC, but not in BCC. The only previously recognized common genetic event in both tumors is *TP53* mutations. We found *TERT* promoter mutations in a substantial proportion of both BCC and SCC. The frequency of these mutations in BCC, SCC, melanoma [19], [20] and other cancers [21] suggests that increased expression of the holoenzyme telomerase is an important event in a wide range of human malignancies.

The role of UV-mediated tumorigenesis in BCC and SCC is supported by epidemiologic data and by the presence of UV-signature mutations in *TP53* (BCC and SCC), *PTCH1* (BCC) or *RAS* (SCC) [2], [3], [5], [11], [16]. The mutations we identified in the *TERT* promoter have a UV-signature with C>T or CC>TT changes, consistent with an etiologic role for UV exposure. However, c.-124C>T and c.-146C>T mutations have also been identified in cancer types such as hepatocellular cancer, bladder cancer, thyroid cancer and gliomas, in which UV-induced mutations are unlikely [21]–[23]. CC>TT alterations are considered virtually pathognomonic of UV-induction [16], [27] and were rare or not described in the aforementioned tumors, however were frequent in cutaneous melanoma and other cutaneous tumors occurring on sun-damaged skin [20], [25]. There is a rare C>T SNP (rs35550267) at position c.-139. To our knowledge no known SNP has been reported at c.-125 (dbSNP). Thus, although we cannot exclude that of the ten CC>TT alterations detected (six c.-138_139CC>TT, three c.-124_125CC>TT and one c.-126_127CC>TT) some, or potentially even all, represent a preexisting C>T variation with an additional C>T mutation, we do believe these alterations, found in 13% of BCC and 18% of SCC, most likely primarily represent UV-exposure tandem mutations, arguing for UV-exposure inducing *TERT* promoter mutations in these tumors. Future larger studies with paired tumor and constitutional DNA should be able to definitively address the role of dipyrimidine mutations in the *TERT* promoter of BCC and SCC.

Matched blood samples of the tumors analyzed in our study were not available, precluding us from directly excluding the presence of germ-line mutations at the mutation hotspots c.-124 or c.-146. However, germ-line mutations at these hotspots have not been observed in various *TERT* promoter mutation studies which compared paired tumor and normal (blood) tissue isolated DNA [19]–[21], [24], [26], nor were they present in the 1000 Genomes database [28]. This makes it almost certain that the mutations detected at these loci in our tumor cohort were somatically acquired.

Functional studies showed that the identified *TERT* promoter mutations cause a 2-4 fold increase in gene expression [19], [20], most likely by introducing ETS transcription binding sites [19]–[21]. Although multiple adjacent nucleotides could acquire UV-induced C>T or CC>TT mutations (Figure 1), the mutations identified in BCC and SCC in our study almost exclusively affected the *TERT* promoter at the described functionally relevant hotspots. This clearly implies a high selection pressure for these mutations, resulting in overexpression of the telomerase holoenzyme. Telomerase expression in tumors is critical for maintaining telomere length and chromosomal stability, which allows cells to continuously proliferate without becoming genetically unstable, and avoiding apoptosis or senescence [29], [30].

The only significant correlation with clinical parameters observed in our cohort, was a slight difference in age between *TERT* promoter mutant and non-mutant BCC. Our cohort is small and larger more detailed follow up studies will be required to verify this finding and to determine if additional clinicopathologic correlations with *TERT* promoter mutation status can be identified.

In summary, our study identifies *TERT* promoter mutations with a UV-signature as frequent events in BCC and SCC non-melanoma skin cancer. Similar results independently validating these findings were recently reported by Scott et al., who found recurrent *TERT* promoter mutations in 78% of BCC and 50% of SCC [26]. Future studies will be required to determine whether *TERT* promoter mutations have prognostic implications or may be targeted therapeutically. This would be especially valuable in patients with metastatic SCC for whom prognosis is poor and effective therapies are lacking.
